# The cost of dementia in an unequal country: The case of Chile

**DOI:** 10.1371/journal.pone.0172204

**Published:** 2017-03-07

**Authors:** Daniel A. Hojman, Fabian Duarte, Jaime Ruiz-Tagle, Marilu Budnich, Carolina Delgado, Andrea Slachevsky

**Affiliations:** 1 Department of Economics, Faculty of Economics and Business, University of Chile, Santiago, Chile; 2 Centre for Social Conflict and Cohesion Studies, Santiago, Chile; 3 Centro KINTUN, Servicio de Salud Metropolitano Oriente, Santiago, Chile; 4 Neurology and Neurosurgery Department, Hospital Clínico Universidad de Chile, Santiago, Chile; 5 Neurology and Neurosurgery Department, Clínica Santa María, Santiago, Chile; 6 Physiopathology Department, ICBM and East Neuroscience Department, Faculty of Medicine, University of Chile, Santiago, Chile; 7 Gerosciences Center for Brain Health and Metabolism (GERO), Santiago, Chile; 8 Cognitive Neurology and Dementia, Neurology Department, Hospital del Salvador, Santiago, Chile; 9 Neurology Service, Medicine Department, Clínica Alemana-Universidad del Desarrollo, Santiago, Chile; 10 Centre for Advanced Research in Education, Santiago, Chile; Universita degli Studi di Perugia, ITALY

## Abstract

We study the economic cost of dementia in Chile, and its variation according to socioeconomic status (SES). We use primary data from a survey of 330 informal primary caregivers who completed both a RUD-Lite and a socio-demographic questionnaire to evaluate the severity of dementia and caregiver’s burden. The costs of dementia are broken into three components: direct medical costs (medical care, drugs, tests); direct social costs (social service, daycare); and indirect costs (mostly associated to informal care). The average monthly cost per patient is estimated at US$ 1,463. Direct medical costs account for 20 per cent, direct social costs for 5 per cent and indirect costs for 75 per cent of the total cost. The mean monthly cost is found to be inversely related to SES, a pattern largely driven by indirect costs. The monthly cost for high SES is US$ 1,083 and US$ 1,588 for low SES. A multivariate regression analysis suggests that severity of dementia and caregiver’s burden account for between 49 and 70 per cent of the difference in the indirect cost across SES. However, between one-third and one-half of the variation across SES is not due to gradient in severity of dementia. Direct medical costs increase in higher SES, reflecting differences in purchasing power, while indirect costs are inversely related to SES and more than compensate differences in medical costs. Moreover, in lower SES groups, female caregivers, typically family members who are inactive in the labor market, mostly provide informal care. The average annual cost of dementia in Chile (US$ 17,559) is lower in comparison to high-income countries (US$ 39,595) and the proportion of cost related to informal cost is higher (74 per cent compared to 40 per cent). SES is a key determinant in the cost of dementia. In the absence of universal access to treatment, part of the social cost of dementia potentially preserves or increases income and gender inequality.

## Introduction

In 2015, 46.8 million people were living with dementia. This number is expected to double every twenty years, reaching 131.5million in 2050. This is mainly because the disease increases exponentially with age, affecting 10 per cent of people over 65 and about 50 per cent of people over 85, worldwide [1], the majority of which are projected to be in low and middle-income countries including Latin America [2]. Crucially, dementia directly affects not only patients but also the caregivers who support them [3, 4]. A high proportion of people with dementia receive care, mostly from family members, ranging from the provision of instrumental activities of daily living, to full personal care and round-the-clock supervision [5].

The economic burden of dementia is of increasing importance given the demographic changes that are leading to dramatic worldwide increases in dementia prevalence. In 2010, the total estimated worldwide cost of dementia was US$ 817.9 billion, roughly one percent of global gross domestic product (GDP). This cost has three components: i) direct costs that include medical expenses (visits, tests, medicines), ii) social costs associated with paid formal caregiving by health professionals or institutionalization, and iii) indirect costs associated with informal caregivers–family members, friends or neighbors–who are unpaid but forgo paid jobs and thereby suffer a productivity loss.

The overall level and composition of the cost of dementia varies widely across countries. In high-income countries, the cost is 1.2 percent of GDP and it is mostly formal. In contrast, in low-income countries it is just 0.24 percent of GDP and most of the costs are informal [6]. These cross-national differences may reflect both demographic changes and marked differences in national spending in health and social protection. While inequality in health has become a salient research and policy issue, as far as we know no study has explored the cost inequalities across different social groups within a country [2].

This paper estimates the costs of the formal and informal care of dementia patients based on data collected from the caregivers of patients who attend primary care centers or memory clinics in the public and private system. It then compares the level and composition of these costs across the socioeconomic status (SES) of the patient. We also characterize differences in the severity of dementia and burden on caregivers across SES groups and assess the extent to which these differences explain the unequal cost of dementia among these groups. We believe this study is particularly relevant in countries with high socioeconomic inequality and relatively weak social protection systems, as is the case for most Latin American countries. Specifically, in spite of more than twenty-five years of stable democracy and sustained economic growth, inequality in Chile has remained high. In 2009, the GINI coefficient was 0.53, right around the average of Latin America. An important expression of inequality is the existence of a two-tier health system, with a private component serving mostly higher income households and a public component serving more than three quarters of the population, with important differences in coverage and quality across components [7]. While the public coverage of medical care has increased significantly since 2000, it does not cover a comprehensive dementia treatment plan that includes caregiving -an essential part of the treatment of this disease. In an unequal country without universal coverage of dementia treatment, if access to formal dementia care is limited by purchasing power it may lead lower SES households to rely more heavily on informal care, forgoing labor productivity. This paper aims to investigate the extent to which socioeconomic inequality translates into differential access to formal care and the patterns of informal care.

## Methods

### Participants

The study is observational, cross-sectional and uses convenience sampling, which is aimed to include urban community-living dyads of patients with dementia and their primary informal caregivers from different settings. An informal caregiver is defined as the person essentially responsible for providing or coordinating the patient’s care needs. Subjects were recruited between March 2009 and June 2011 and were contacted through acting physicians from primary care centers, secondary level neurological consultations and caregiver support groups.

The inclusion criteria for the patient were: i) Clinical history of a diagnosis of dementia; ii) a score ≥ 57 in the Short Spanish version of the Informant Questionnaire of Cognitive Decline in the Elderly (SS-IQCODE) [8]; and iii) presence of a primary, unpaid caregiver who has known the patient for a minimum of 10 years (the SS-IQCODE evaluates changes over a decade) and meets with the patient at least once a week.

### Study design and assessments

The CUIDEME study survey is based on a self-completion questionnaire using clinically validated scales, informant-based assessments of patient characteristics and self-report questionnaires to measure caregiver variables [9]. An initial open-ended interview was used to establish that the person recruited for the study was most directly responsible for providing care. After obtaining informed consent, the caregivers were asked to answer self-completion questionnaires to collect the following data:

- Socioeconomic status (SES). We classified socio-economic status based on the European Society for Opinion and Marketing Research (ESOMAR) definitions, which classifies households into five social levels [10, 11]. The first level (AB) corresponds to high SES, the next three levels (CA, CB, D) represent middle SES groups, and the last level (E) designates low SES. In order to obtain reliable mean estimates of the patients and caregiver variables for each SES category, we combined the patients of the two lowest SES levels into a single group. Hence, the SES classification used in this paper has four groups: SES 1 (D and E households) is the *lowest group*; SES 2 (CB households) is the *lower-middle group*; SES 3 (CB households) is the *upper-middle group*; and SES 4 (AB households) is the *highest group*. Table 1 shows the share of the adult population (aged 18 and over) and the annual household income per capita for each SES.
  For robustness, we repeated our estimates using a different definition of the SES group using exclusively education levels. A summary of these results, which confirm the ones presented shortly, is available in S1 Tables.
- Confirmation of a diagnosis of dementia. The caregiver was asked to complete the SS-IQCODE survey, which is a validated 16-question instrument that assesses the patient’s memory and ability to function independently. It is used to confirm a diagnosis of dementia [8]. This survey was used alongside a clinical characterization of dementia through two further questionnaires: the Activities of Daily Living Questionnaire (ADLQ) [12], which assesses functional impairment; and the Neuropsychiatric Questionnaire (NPI-Q) [13], which assesses the presence of neuropsychiatric symptoms.
- Caregiver burden. We used the Zarit Burden Interview (ZBI), validated for Chile [14], and the General Health Questionnaire (GHQ-12) [15], to assess the presence of psychiatric morbidity in caregivers.
- The cost of dementia. We estimated this cost using the Resource Utilization in Dementia lite (RUD-lite) instrument, a validated questionnaire broadly used in the literature [16]. Caregivers were asked to recall the resources used during the last month, broken down into direct and indirect costs. Direct costs refer to money explicitly exchanged for health costs or heath care resources (hospital, medical services, drugs), and social care costs (community care services, special accommodation or social services). Indirect costs refer to money implicitly by way of a loss of income by the patient and/or loss or reduction of income by family members or informal caregivers depending on the number of hours that informal caregivers spend with patients, such as friends, depending on the time they spend with the patient without monetary reimbursement. This type of cost is also referred to as informal care costs, i.e., the time spent by the primary non-professional caregiver on the personal and instrumental activities of daily living (ADL) tasks and supervision. To measure informal care, we used caregiver’s reports on the amount of time spent on personal and instrumental ADL, and supervision. They could not report more than 24 hours per day spent on each informal care task, although the sum across tasks could be more than 24 hours [17, 18].

**Table 1:** Annual Household income per capita and population share by socioeconomic status*.

	Population Share	Average per capita Income^i^
	%	US$
SES 1^ii^	43.3	5,456
SES 2 ^ii^	29.9	6,899
SES 3 ^ii^	13.0	10,968
SES 4 ^ii^	13.7	23,633

*Authors’ calculation based on the 2011 National Socioeconomic Characterization Survey.

^i ^Annual household income per capita expressed in PPP US dollars.

^ii ^SES 1–4 label four socioeconomic group levels ordered from lowest to highest.

### Cost calculation

The costs per person were separated according to resource consumption:

TotalCosts=(MedicalCareCosts)+(SocialCareCosts)+(InformalCareCosts)

Each component of the cost is calculated by multiplying the unitary cost of each resource by quantities of resources used. The unitary prices used for medical and social care calculations were based on market averages. A summary of these unit prices can be found in S2 Tables

For robustness we present three different estimates of informal costs. In the literature, there are different approaches to calculate informal care costs, with different positive and normative implications. As argued by Wimo et al. (2000, 2010) [17, 18] and others ([19–26]) “costing informal care is complicated and controversial.” The two main approaches are the replacement cost approach and the opportunity cost approach (sometimes referred as productivity loss). Replacement cost refers to the cost associated to replacing informal care by formal care. This estimate is typically implemented using a reference salary and multiplying this salary by the informal care hours reported. The salary used varies across studies. It is either the average salary of a formal caregiver or a minimum wage salary (which simplifies comparisons as it is widely available for most countries). We present estimates for both of these cases. We use the legal minimum wage for the year 2009.

The productivity loss approach aims to capture the opportunity cost of non-working time. The implementation varies across studies depending on the information available. Studies specify different working hours and salaries, and differ on how they treat caregivers with ages above the retirement age. In line with recent studies, using the education level and age of the caregiver, we estimated a Mincer salary equation to estimate the salaries of caregivers younger than 65 years of age (below the legal retirement age). This estimation was done using the National Socioeconomic Characterization Survey (CASEN), the main household survey in Chile used for policy and labor market evaluation (a representative sample of more than 70,000 households). The salaries are used to value the standard legal limit of 45-hours-a-week labor workdays. For caregivers aged 65 or more, we used the average caregiver salary, which amounts to a replacement cost for caregivers in this age group. The average Mincer wages for each SES group can be found in On-line Appendix 1.

All costs are expressed in PPP US dollars to facilitate comparisons. Local currency values were converted to dollars using average PPP exchange rate in 2009 –the year the survey was conducted. The PPP exchange rate was obtained from the official World Bank database. This rate was 353.2 Chilean pesos per US Dollar.

### Statistical analysis

We used STATA 13.1 for the univariate and multivariate statistical analyses. A general linear regression model (GLM) with log link function was used to study the factors that explain the direct and indirect costs of dementia. These factors included measures of the severity of the patient’s condition, the caregiver’s burden and patient’s SES.

### Ethics

All the participants gave their written informed consent. The ethics committee of the Metropolitan Health Service approved the study protocol.

## Results

We recruited 387 dyads of unpaid caregivers and patients with dementia. Fifty-seven caregivers (14.9 per cent of the initial sample) were excluded because their patients’ SS-IQCODE scores were below 57 points. The total number of dyads satisfying inclusion criteria was 330. The average age of patients and, caregivers was 76.78 (range 46–103, SD: 10.11) and 60.75 (range 20–91, SD: 13.61), respectively (see S1 Dataset). The share of patients receiving health care for dementia in the public system was 56.4 per cent and the remaining 43.6 per cent in the private system. Patients attending the public system attend either secondary care facilities (47.9 per cent) and/or primary care facilities (38.5 per cent). The main socio-demographic and clinical characteristics of dementia patients and primary caregivers in our study are presented in Table 2.

**Table 2:** Socio-demographic and clinical characteristics of the patients and caregivers*.

	Patient	Caregiver
Female	62.5	74.3
Male	37.5	25.6
20 to 60 years old	5.7	46.4
61 to 80 years old	55.5	47.0
80 + years old	38.8	6.6
Primary Education	47.0	16.6
Secondary Education	26.2	39.0
Tertiary Education	26.8	47.3
SES 1 ^i^	38.7	.
SES 2 ^i^	37.7	.
SES 3 ^i^	14.8	.
SES 4 ^i^	8.7	.
Public Health System	5.8	.
Private Health System	67.7	.
Other Health System	16.2	.
Observations	328	328

* Results are expressed in percentage (%).

^i ^SES 1–4 label four socioeconomic group levels ordered from lowest to highest. There are two observations that do not have the age of the caregiver.

As shown in Table 2, our sample of patients contains more women (63 per cent), in part due to gender differences in longevity. Not surprisingly, 94 per cent of the sample are aged 60 and over. Accordingly, the sample of caregivers is younger and more educated. Almost three-quarters of caregivers are women. The two lower SES groups, SES 1 and SES 2, comprise three quarters of the patients’ sample, which coincides with the combined weight of these groups in the Chilean adult population. Patients in the middle-upper SES 3 group and the highest SES 4 group make up 15 and 9 per cent of the sample, respectively, compared to 13 and 14 per cent for each group in the overall adult population (see Table 1).

The survey does not include household income data. The CASEN survey is a nationally representative household survey that contains not only all of the variables in our survey that were used to define the SES groups but also individual-level income information. Using the 2011 CASEN we calculated the household per capita income of each SES group, which serves as a benchmark for the costs of dementia. This information, summarized in Table 1, is useful to visualize the material inequality across groups. Note that the income difference between SES 1 and SES 2 is relatively small and that the average income of group SES 4 more than doubles that of SES 3.

Table 3 summarizes the correlation of job opportunity loss for caregivers, disaggregated by gender and age. Detailed accounts of the formal and social components are available upon request. Note that 47 per cent of caregivers are younger than 60 (implying a high labor opportunity cost). In this group, roughly one quarter declared they were not working due to caring responsibilities (28 per cent of women caregivers and 29 per cent of men), and more than 67 per cent said they would look for a job if they did not provide care. These numbers fall to 16 per cent and 44 per cent respectively if caregivers of all ages are considered.

There are important gender differences among caregivers. Aside from the fact that 74 per cent of informal caregivers are women (Table 2), nearly half of the women caregivers are less than 60 years old (the legal retirement age for women in 2009). In contrast, only one-third of the male caregivers are in this age bracket (Table 3).

**Table 3:** Caregiver’s loss of job opportunity by gender and age*.

	Women			Men				All		
	18–60	61 and +	All	18–60	61 and +		All	18–60	61 and +	All
Working (%)	43	18	30	63		25	38	47	20	32
Not working due to care (%)	28	10	17	33		13	16	29	11	16
Loss due to care (days per month)	3 (7)	2 (10)	3 (8)	3 (4)		3 (7)	3 (6)	3 (6)	3 (9)	3 (7)
Loss due to care (hours per day)	5 (8)	4 (8)	4 (8)	6 (7)		7 (10)	6 (9)	5 (8)	5 (9)	5 (8)
Search job if not to care (%)	66	32	46	70		31	39	67	32	44
N of Observations	114	121	235	27		52	79	141	173	314

*The values for days per month and hours per day lost due to care correspond sample means. Standard errors in parenthesis.

The estimation of indirect costs in Table 4 is computed using the replacement cost method with a minimum wage. All values are in PPP US dollars. By construction this method yields the most conservative estimates of the cost of dementia (see Table 5). Table 5 summarizes three stylized facts about the mean annual total cost of care and its components. First, for the whole sample, the total cost is $17,559, of which 75 per cent corresponds to indirect costs, 20 per cent to direct medical costs and only 5 per cent to direct social costs.

**Table 4:** Mean annual cost of care by socioeconomic status*. Indirect cost = replacement with minimum wage

	Hours per month devoted to patient care	Direct Medical Cost	Direct Social Cost	Indirect Cost	Total
	Mean	US $	US $	US $	US $
SES 1^i^	387	3,111	652	15,277	19,050
SES 2^i^	345	3,451	833	13,626	17,919
SES 3^i^	247	3,787	1,874	9,758	15,426
SES 4^i^	200	4,288	796	7,902	12,991
All	334	3,442	914	13,194	17,559

* Indirect Costs is estimated using the replacement cost of a caregiver imputing a minimum wage.

^i^Results are mean values for each SES group (PPP values). SES 1–4 label four socioeconomic group levels ordered from lowest to highest.

**Table 5:** Annual indirect cost of care by socioeconomic status with different methods*.

	Replacement 1 Minimum wage	Replacement 2 Avg. caregiver wage	Productivity Loss Mincer wage 18–64 Avg. caregiver wage 65+
	US $	US $	US $
SES 1^i^	15,277	22,350	23,952
SES 2^i^	13,626	19,935	23,503
SES 3^i^	9,758	14,276	17,490
SES 4^i^	7,902	11,560	16,362
All	13,194	19,302	22,164

Each column uses different estimates of Indirect Costs. Replacement 1 and 2 columns use the replacement cost of a caregiver imputing a minimum wage and the average wage of a caregiver in Chile, respectively. The Productivity loss column estimates the wages lost by caregivers less than 65 years old, and the cost of a caregiver for those aged 65 and older.

^i ^Results are mean values for each SES group (PPP values). SES 1–4 label four socioeconomic group levels ordered from lowest to highest.

**Fig 1:**
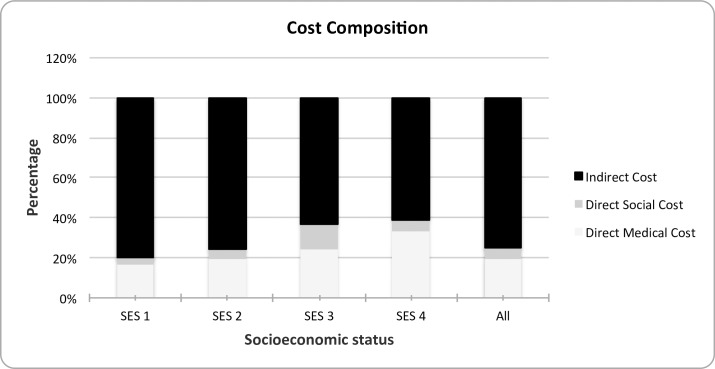
Distribution of cost by socioeconomic status. SES 1–4 label four socioeconomic group levels ordered from lowest to highest.

Second, there are important differences across socioeconomic groups. The total cost decreases as we move up the socioeconomic ladder. Indeed, the cost of dementia for the bottom SES group is 47 per cent higher than that of the highest SES group. The relative importance of each of the cost components is shown in Fig 1.

Table 5 provides the estimates of indirect costs using the three methodological approaches discussed earlier. The first column coincides with the third column in Table 4, the second one uses the replacement cost method using the average caregiver salary which is 46 per cent higher than the minimum wage (yielding a total average indirect cost of US$ 19,302 dollars rather than 13,194). The third column uses the opportunity cost approach: Mincer wage estimates by age and education level for caregivers younger than 65 years and a replacement cost using an average caregiving salary for those 65 and older. The average indirect cost is now roughly 9,000 dollars larger than the first column. Regardless of the method, the direction of SES gradient remains the same, although it is less steep using the productivity loss method. The percentage differences between low and high SES are smaller with that third method, reflecting the fact that higher SES have larger salaries.

Table 6 shows that the burden and psychological distress on informal caregivers is considerable, in line with previous studies on dementia caregiving [19]. Indeed, as 67.6 percent of caregivers have severe overload (Burden, ZBI score > 55). In addition, all the measures display a marked socioeconomic gradient. The percentage of caregivers with severe overload for patients in the lowest SES group is 73 per cent, 23 percentage points higher than the top SES group (the average difference in caregiver burden between SES 1 and SES 4 is 0.65 of a standard deviation). While the percentage of caregivers with some mental health disorder (Psychiatric morbidity, GHQ-12 score>4) is lower, reaching 47 per cent, the difference across the SES 1 and SES 4 groups is large as well (0.28 of a standard deviation).

The same pattern holds for patients. Lower SES is associated with significantly more functional impairment as measured by the ADLQ score (the average difference between SES 1 and SES 4 is 13.35 points, 0.54 of a standard deviation) and neuropsychiatric symptoms as measured by the NPI score (the average difference between SES 1 and SES 4 is 2.18 points, two thirds of a standard deviation).

**Table 6:** Caregivers and patients wellbeing by socioeconomic status*.

	Caregiver				Patients			
	ZBI^i^	Burden^ii^	GHQ-12^iii^	Psychiatric Morbidity^iv^	Functional Impairment^v^	Neuropsychiatric symptoms^vi^	Antidementiadrugs^vii^	Paid Caregiver^viii^
SES1^ix^	63.82±17.88 (0–104)	73.0%	3.87±2.85 (0–12)	49.2%	67.8±22.73 (7–100)	6.79±3.21 (0–12)	34.1%	14.3%
SES2 ^ix^	61.69±18.81 (0–100)	64.6%	3.85±3.05 (0–12)	50.4%	60.12±25.2 (7–100)	6.01±3.28 (0–12)	51.2%	19.7%
SES3 ^ix^	60.73±15.52 (0–102)	71.4%	3.31±2.33 (0–9)	44.9%	60.08±24.54 (0–98.81)	5.59±3.06 (0–12)	71.4%	32.7%
SES4 ^ix^	52.18±14.8 (24–87)	50.0%	2.57±2.18 (0–9)	28.6%	54.45±29.41 (0–98.81)	4.61±2.99 (0–10)	71.4%	21.4%
Total	61.55±17.88 (0–104)	67.6%	3.67±2.83 (0–12)	47.3%	62.5±24.87 (0–100)	6.12±3.25 (0–12)	49.4%	19.7%
N. of Obs.	328				328			

* Results are expressed in mean ± standard deviation (minimum–maximum).

^i ^Score on the Zarit Burden Interview (ZBI).

^ii ^Percentage of caregivers with severe overload (ZBI> 55).

^iii ^Score on the General Health Questionnaire (GHQ-12).

^iv ^Percentage of caregivers with mental health disorder (GHQ-12)≥ 4).

^v ^Score on the Activities of Daily Life Questionnaire (ADLQ).

^vi ^Score on the Neuropsychiatric Inventory Questionnaire (NPI).

^vii ^Percentage of patients using an specific antidementia drugs (anticholinesterasic and/or memantine).

^viii ^Percentage of patients with a paid caregiver.

^ix ^SES 1–4 label four socioeconomic group levels ordered from lowest to highest.

### Regression analysis

We use a Generalized Linear Model (GLM) with log link to analyze some of the potential determinants of the cost of treatment. The sample size is lower than the original because there are missing values for some of the main variables. The explanatory variables of our multivariate analysis include demographic characteristics (age and a gender indicator); measures that characterize the neuropsychiatric condition of the patient (NPI) and their functional impairment (ADLQ), and measures of some of the resources available for the treatment, including an indicator if the individual has a caregiver, the ZBI burden index of the caregiver and the patient’s SES (SESD 1 is an indicator that takes a value of one if the patient is in SES 1 and zero, otherwise; similarly for SESD 2, SESD 3, and SESD 4).

The results of this estimation are presented in Table 7. We find that cost rises with age, although these coefficients are not statistically significant. There is no significant gender association. The coefficient of the caregiver burden measure, ZBI, is statistically significant. The two measures of the severity of the symptoms are positively associated with the total cost, although only ADLQ is statistically significant. While having a paid caregiver is associated with a lower cost, it is not statistically significant. None of the SES indicators is significant. The lack of a statistical significance between total costs and SES shown in Table 7 is consistent with the fact that SES is positively correlated with formal costs but negatively correlated with informal care, as confirmed by the negative and significant coefficient shown in Table 8 below.

**Table 7:** Determinants of the total cost. Regression analysis of total annual costs of care (GLM model with log link).

	Observed	Bootstrap			95% Conf. Interval	
Depvar: Total Cost	Coef.	Std. Err.	Z	P>z	Lower	Upper
Patient's Age	-0.002	0.004	-0.34	0.731	-0.010	0.007
Patient Male = 1	0.110	0.095	1.16	0.247	-0.076	0.297
ZBI^i^	0.004	0.003	1.47	0.141	-0.001	0.010
NPI-Q^ii^	0.033	0.015	2.18	0.029	0.003	0.062
ADLQ^iii^	0.013	0.003	4.93	0.000	0.008	0.018
Paid caregiver ^iv^	-0.132	0.114	-1.16	0.248	-0.356	0.092
SES1^v^	0.138	0.166	0.83	0.404	-0.187	0.464
SES2^v^	0.147	0.167	0.89	0.376	-0.179	0.474
SES3^v^	0.067	0.207	0.33	0.744	-0.338	0.473
Constant	11.816	0.377	31.36	0.000	11.078	12.555
N of Observations	306					

Notes

^i^ Zarit Burden Interview (ZBI).

^ii ^Neuropsychiatric Inventory Questionnaire (NPI-Q).

^iii^Activities of Daily Life Questionnaire (ADLQ).

^iv^ Availability of a paid caregiver.

^v^ SESD 1–3 are indicator variables for SES levels 1,2 and 3, respectively. SESD 4 is omitted. Bootstrap standard errors with 1,000 replications.

**Table 8:** Determinants of the indirect costs. Regression analysis of indirect costs of care (GLM model with log link).

	Observed	Bootstrap			95% Conf. Interval	
Depvar: Indirect Cost	Coef.	Std. Err.	z	P>z	Lower	Upper
Patient's Age	-0.009	0.007	-1.37	0.172	-0.023	0.004
Patient Male = 1	-0.063	0.143	-0.44	0.658	-0.344	0.217
ZBI^i^	0.008	0.005	1.60	0.110	-0.002	0.018
NPI-Q^ii^	0.051	0.022	2.31	0.021	0.008	0.094
ADLQ^iii^	0.023	0.004	5.67	0.000	0.015	0.031
Paid caregiver ^iv^	-0.302	0.176	-1.72	0.085	-0.647	0.042
SES1^v^	0.611	0.262	2.34	0.020	0.098	1.124
SES2^v^	0.560	0.271	2.06	0.039	0.028	1.091
SES3^v^	0.371	0.312	1.19	0.233	-0.239	0.982
Constant	10.719	0.713	15.04	0.000	9.322	12.116
N of Observations	306					

Notes

^i^ Zarit Burden Interview (ZBI).

^ii^ Neuropsychiatric Inventory Questionnaire (NPI-Q).

^iii^Activities of Daily Life Questionnaire (ADLQ).

^iv^ Availability of a paid caregiver.

^v^ SESD 1–3 are indicator variables for SES levels 1,2 and 3, respectively. SESD 4 is omitted. Bootstrap standard errors with 1,000 replications.

Since indirect costs represent nearly three quarters of the overall costs of dementia and are responsible for most of the cost variation across the different SES groups, we present the same estimation using total indirect cost as the dependent variable. The results are summarized in Table 8. As before, the patients’ symptom severity measures NPI-Q and ADLQ are statistically significant conditional correlations (at 2 and 1 per cent significance, respectively) and positively affect the cost. In contrast to the estimates for total costs (Table 7) the caregivers’ burden measured by ZBI and the coefficients of the two lowest SES indicators are now significant and positively related to the indirect cost. The variable indicating the use of paid caregivers is negatively related to cost, consistent with the hypothesis that formal care can substitute the informal care captured by the indirect cost.

From Table 5, we see that lower SES levels are associated with higher severity of functional impairment (ADLQ) and neuropsychiatric symptoms (NPI-Q). It is natural to ask whether the additional costs of lower SES patients–mostly associated to more hours of caregiving–are explained by these differences in symptoms severity. Using the estimates from the regression in Table 8, we find that 70.4 per cent of the difference in the indirect cost of the highest and lowest SES patients is explained by differences in the patients’ symptoms severity (The average difference in ADQL between SES 1 and SES 4 patients is Δ*ADLQ* = 67.80 − 54.45 = 13.35, and, similarly, the difference in NPI is Δ*NPI* = 6.79 − 4.61 = 2.18. From the generalized linear model with log link function estimated in Table 8, the percentage change in the indirect cost associated to these differences in symptoms’ severity across groups is 1 − *e*^−(0.023Δ*ADLQ*+0.051Δ*NPI*)^ = 0.342. The overall percentage difference between these groups is 0.486. The ratio between these two numbers is 70.4 per cent). If we collapse the two lowest SES groups into one (SES 1 and SES 2) and do the same with the two highest ones (SES 3 and SES 4), 48.6 per cent of the difference in indirect costs are solely explained by the higher symptom severity of lower SES patients.

## Discussion

This study uses primary data from a survey of 330 Chilean dementia patients and caregivers to estimate the annual costs of dementia and how they vary across SES groups. Most studies on cost of dementia with larger samples are from high-income countries [In the USA (8,671 patients and the subsample of individuals with dementia of the 10,903 participants of the Health and Retirement Study); Italy (423 patients); Spain (869 patients and 560 patients); Korea (604 patients); Belgium (386 patients); UK, France, Germany (419 patients in France, 552 patients in Germany and 526 in the UK)] [27–34], with the exception of the studies using data from seven low and middle-income countries (LMICs) reported in the World Alzheimer Report 2015 (11 sites, n = 15,022)] [2, 35]. The cost of dementia and its distribution varies according to country income. Although the cost of dementia is larger in higher income countries, there are no studies focused on cost differences within the same country [2, 20, 23].

There are five main issues regarding the results we obtained that are important to discuss: the level of the average cost of dementia that we computed; the composition of this average cost in direct and indirect cost; the variation of the cost across socioeconomic status (which is something new within the literature); how the composition and this gradient are compared with results regarding severity of dementia; and finally the implications these results hold for policymakers.

First, we find that the average cost is about US$ 17,500 (PPP), less than half of the estimates for European countries. This cost is higher than the cost reported in other Latin American countries. In Argentina, for example, a study using a sample of 105 patients determined that the direct cost of dementia ranged from US$ 3,420 to US$ 9,657 depending on the severity of the illness and the type of dementia [36]. In another study for Brazil, it was reported that direct costs of dementia are equal to 66 per cent of family income (approximately US $300 in their sample) [37]. On the other hand, in Peru, for 106 patients attending a private clinic, the cost was estimated at US$ 6,844 per year [38]. Liu (2013, unpublished) [35], reported a higher cost in people of the public level (US$ 6,750) compared to the private level (US$ 1,887), both costs calculated at international dollars [26]. The total cost of dementia in our study is slightly higher than the extrapolation of cost would be from cross-national regressions [2]. Wimo, Winblad & Johnson [39, 40] estimated an overall cost of US$ 13,194 for Latin-America.

We should be cautious to extrapolate the cost from our results to all dementia patients since our sample comprises only subjects diagnosed with dementia. Other studies show that roughly one half of those who would meet the diagnostic criteria for dementias have been diagnosed [41, 42, 43]. The cost of dementia is higher in patients with a diagnosis of dementia compared to population-based studies, probably because the rate of diagnosis is higher in patients more severely impaired [27, 28]

Thus, external validity of our results could be limited by the fact that our sample is one of convenience. It is hard to assess whether our sample is representative of the population of dementia patients in Chile as there is no epidemiologic study of dementia in Chile. The closest proxy is the National Survey of Dependency in the Elderly (NSDE)[44], representative of the Chilean elderly population. While the NSDE uses different tests (a subject is classified with dementia if he/she is over 60 years of age, scores below than 13 points on the MMSE scale and above 5 points on the Pfeiffer scale) to screen for dementia than the ones we have, the tests measure similar symptoms and impairment but are not fully comparable. The proportion of female patients with dementia in the NSDE is 60.7 per cent, a number quite close (statistically the same) to the proportion of 62.9 per cent in our sample. We also find that the average age of patients with dementia in the NSDE for patients over 60 years, the average age is 80.2 ± 8.7 years. If we truncate our sample to patients of age 60 years or older, the average age is 78.7 ± 9.7 years, which is not statistically different.

In sum, at least in terms of the NSDE and basic demographics such as gender and age, our sample does not show significant differences with population samples. Finally, as discussed previously (see Table 1) the SES distribution in our sample is quite similar to the distribution for the entire population of the country. This suggests that, at least in the SES dimension, which is central in our paper, our data is consistent with country’s population. Nonetheless, we caution about the fact that our estimates should not be extrapolated to the entire population of patients with dementia in Chile. Instead, they are likely to be valid cost estimates for patients who received a formal diagnosis of dementia.

Second, regarding the composition of the total cost, in contrast to advanced democracies, indirect costs make up a large proportion of the overall cost (74 per cent) and it is related to the productivity loss of informal caregiving, mostly borne by a female family member (as in low-income countries). The distribution of cost differed from the estimation of composition (direct versus indirect costs) from country income per capita. Using cross-national regressions that relate cost composition to GDP, Wimo, Winblad & Johnson [39] predict for Latin America that direct costs comprise US$ 4,943 and indirect costs US$ 8,974. Our estimates for Chile, yield considerably lower direct costs and higher indirect costs, hence, a larger contribution of indirect costs to the total (75 per cent versus 64 per cent). These results could be explained by the lack of developed health services that can meet the needs of patients with chronic, non-transmissible diseases such as dementia [45–48].

Third, a large body of evidence suggests that socioeconomic determinants, such as disparities in income, education, occupation, and other dimensions of SES, account for appreciable variance in all cause and disease-specific morbidity and mortality rates, as well as the prevalence of risk factors for chronic medical conditions [49–51]. Within-country inequity has also emerged as key determinant of health [52]. Previous data in Chile suggested that in the elderly population, disability and mortality is higher in low socioeconomic groups [53]. In line with this evidence, we found marked socioeconomic gradients, the severity of the patients’ symptoms and the psychological burden on caregivers were negatively correlated with SES. Several reasons could be associated with this difference. First, the rate of progression of dementia could differ across SES, although the factors related to progression of dementia are not clear. Indeed, higher educational level is related to rapid cognitive decline in dementia [54]. On the other hand, patients with dementia in higher SES groups have better access to formal care but the effect of formal care in delaying progression of dementia is not well established. For example, the effectiveness of drug treatment remains controversial [55]. More generally, the higher severity of dementia in lower SES groups is consistent with evidence that shows a strong correlation between socioeconomic status and health outcomes [49, 50]. When it comes to costs, while high SES groups spend more on formal care, an inverse correlation holds for indirect cost. The indirect cost for the lowest SES group is twice as large as that of the highest SES group. Since most of the cost is indirect, the average total cost is higher for lower SES groups. The larger amount of informal cost in low SES could reflect social segregation in access to health care [51, 52]. Additionally, we cannot exclude that the asymmetry of direct and indirect cost across SES groups is partly due to differential quality of care between formal and informal care. Nevertheless, we are unaware of any study comparing the efficiency of formal versus informal care in dementia.

Third, between one-third and one-half of the variation of informal explained by SES is not due to the gradient in severity. This difference could be associated with differences in the education of caregivers or patients that directly affect the efficacy of the treatment, or a contextual factor, which both require further investigation. To interpret the results it is important to note that, in the last decade, Chile was among a few Latin American countries to enter the group of high-income countries. Still, as is the case in most of the region, it remains a highly inequitable country with marked social differences in access to private health care consumption and primary goods such as health [56]. The Chilean health system is characterized by the coexistence of two insurance systems, a subsidized public system (FONASA) that covers nearly 80 per cent of the population and a private system (ISAPRE) that covers mostly middle-high and high-income households. Thus, a manifestation of socioeconomic inequality is the significant socioeconomic segregation in the health system. Since the country lacks public policies for dementia [57], lower SES dementia patients cannot afford formal treatment and routinely resort more to informal care. This induces a lower quality of dementia treatment and a higher productivity loss for low SES informal caregivers–typically family members- relative to higher SES patients. In the absence of universal care for dementia, this disease is likely to amplify socioeconomic inequality.

Finally, at the same time, Chile (similarly to other countries in Latin America) has low female participation in the labor-market (45 per cent) and a gender-salary gap bordering 30 per cent. The indirect costs of dementia are associated with the caregivers’ loss of job opportunities and have a gender bias. As found in previous studies, women assume most of the informal care cost because the vast majority are women [36, 58]. Moreover, the cost of productivity loss is higher in women. The majority of women caregivers are aged under 60 and one-quarter expressed that they did not work due to their care burden. Thus, dementia care contributes to gender inequality. As demographic trends increase the share of elderly in the population, the contribution to gender inequality associated to dementia care could grow over time.

In light of previous studies, we think the paper has three main contributions. First, there are only a handful of studies in Latin America that study the cost of dementia with a relatively large sample of patients. Indirect costs make up the main component of dementia costs in Chile, which is not dissimilar to most developing countries.

Second, we know of no studies of dementia that look at the gradient of dementia costs across SES within a country. The closest is perhaps is Liu (2013, unpublished) [2, 35] who estimates costs for patients with dementia in the private and public health systems in low income countries (if lower SES patients are more likely to be treated in the public than in the private system, the system serves as an indirect proxy of SES).

Third, we find that lower SES patients are associated with higher costs due to informal care and, possibly, symptom severity. This gradient is not obvious ex ante in light of cross-country differences in the cost of dementia, where the cost is higher (PPP) in high-income countries, suggesting that availability of formal care for dementia is a key determinant of cross-country differences in cost. Studying within-country differences across SES groups, as we do for Chile, complements this view. Our results suggest that, in the absence of universal (public) coverage of dementia treatment, underlying inequalities lead to unequal access to formal care (as formal care is market-based and increases with income). This leads lower SES households to spend more time on informal caring activities, the main driver of differences in costs across SES in our study. This does not necessarily hold for other diseases, as caregiving needs are especially high for dementia patients.

From a policy perspective, we conclude that, in the absence of universal access to treatment, part of the social cost of dementia is that it can contribute to, preserve or increase income and gender inequality. In summary, our study confirms the importance of in-country inequality in the burden of disease [52]. The study shows that in an unequal country with weak social protection for dementia patients, the economic costs of this disease are sizeable and contribute to socioeconomic and gender inequality. In a country with relatively high income per capita, high wealth inequality and relatively low social insurance, expanding social protection to incorporate formal dementia care would not only contribute to health equality but also reduce socioeconomic inequality by substituting informal care with formal care. This would in turn improve the participation, matching and productivity in the labor market for a population of caregivers that currently do not access formal care. The additional coverage costs to the public sector may well be outweighed by the health and productivity gains.

## Supporting information

S1 Dataset Data used in all analyses. (DTA)

S1 Tables Estimates of costs according to education levels. (DOCX)

S2 Tables Unit prices for medical and social care. (DOC)
