# Supplementary material for: The cost of dementia in an unequal country: The case of Chile
Source: PLoS One. 2017 Mar 7;12(3):e0172204. doi: 10.1371/journal.pone.0172204 (PMC5340351; doi:10.1371/journal.pone.0172204)
Supplement: S1 Tables — (DOCX) [file pone.0172204.s002.docx]

**S1 Tables**

Table A.1 summarizes the age, schooling years, Mincerian hourly wage estimates (average fitted values) and the number of hours of declared by informal care-givers for each SES category.

**Table A.1 Mean Age, Schooling, Mincerian Salary and Informal Care Hours by Socioeconomic Status (Education Level)**

|  | **Age** | **Schooling** | **Mincer**  **Salary** | **Monthly Informal Care** |
| --- | --- | --- | --- | --- |
| **SES** | **years** | **years** | **Ch $/hour** | **Hours** |
| **SES1** | 60 | 11 | 2,393 | 387 |
| **SES2** | 61 | 13 | 3,148 | 345 |
| **SES3** | 62 | 15 | 3,394 | 247 |
| **SES4** | 62 | 16 | 4,046 | 200 |
| **All** | 61 | 13 | 2,928 | 334 |

The following tables parallel tables 4 and 5 in the main text using the patients’ education level to define socio-economic status. Three different education levels are considered: Primary School, Secondary School and Tertiary Education. In Chile, Primary School includes grades 1 through 8, while Secondary School includes grades 9 through 12. Individuals in the Primary School category have at most completed 8^th^ grade, while those in the Secondary School category have a completed at least 9^th^ grade and at most 12^th^ grade. Tertiary Education considers any individual who graduated from high school and completed at least one year of tertiary education.

**Table A.2 Mean Annual Cost of Care by Socioeconomic Status (Education Level)**

|  | **Direct Medical Cost** | **Direct Social Cost** | **Indirect Cost** | **Total** |
| --- | --- | --- | --- | --- |
| **SES**  **Education level** | **US $** | **US $** | **US $** | **US $** |
| **Primary** | 2,138 | 190 | 18,766 | 21,107 |
| **Secondary** | 3,309 | 1,257 | 13,014 | 17,589 |
| **Terciary** | 4,063 | 926 | 11,139 | 16,135 |
| **All** | 3,442 | 914 | 13,194 | 17,559 |

Results are mean values for each SES group (PPP values). Socioeconomic groups based on education levels.

**Table A.3: Annual Indirect Cost of Care by Socioeconomic Status (Education Level) with Different Methods***

|  | Replacement 1  Minimum wage | Replacement 2  Avg. caregiver wage | Productivity Loss  Mincer wage 18-64  Avg. caregiver wage 65+ |
| --- | --- | --- | --- |
| **SES**  **Education level** | US $ | US $ | US $ |
| **Primary** | 18,766 | 27,455 | 27,455 |
| **Secondary** | 13,014 | 19,040 | 19,511 |
| **Terciary** | 11,139 | 16,296 | 22,206 |
| **All** | 13,194 | 19,302 | 22,164 |

Results are mean values for each SES group (PPP values). Socioeconomic groups based on education levels.

Each column uses different estimates of Indirect Costs. Replacement 1 and 2 columns use the replacement cost of a caregiver imputing a minimum wage and the average wage of a caregiver in Chile, respectively. The Productivity loss column estimates the wages lost by caregivers less than 65 years old, and the cost of a caregiver for those aged 65 and older.
