# Supplementary material for: The cost of dementia in an unequal country: The case of Chile
Source: PLoS One. 2017 Mar 7;12(3):e0172204. doi: 10.1371/journal.pone.0172204 (PMC5340351; doi:10.1371/journal.pone.0172204)
Supplement: S2 Tables — (DOC) [file pone.0172204.s003.doc]

**S2 Tables**

The tables in this appendix show the main prices used in the estimation of the direct (drugs and formal care services) and indirect costs of dementia. Table A.1 shows the average prices for the drugs most commonly used by patients and caregivers in the study.

**Table A.1: Drug** Prices

|  |  |
| --- | --- |
| **Name/Type** | **Price (US$)** |
| Antidemencial | 91.27 |
| Antidiabetics | 0.51 |
| Antihypertensive | 3.10 |
| Aspirin | 3.04 |
| BB propranolol | 2.81 |
| Bromazepan | 0.67 |
| Diuretic | 0.88 |
| Statins | 5.07 |
| Escitalopram/Fluoxetine | 4.52 |
| Levothyroxin | 2.03 |
| Calcium | 8.22 |

Source: Author’s calculations based on SERNAC

database and two private pharmacies prices[[1]](#footnote-2)

Table A.2 shows the average prices for the most common health related services used by patients. Finally Table A.3 shows the average of other prices used in the estimation.

**Table A.2: Health related services p**rices

| **Name/Type** | **Price (US$)** |
| --- | --- |
| Health Center | 3.28 (daily) |
| Ambulance | 53.67 (6.2 miles) |
| Hospital day | 49.86 |
| Physician | 13.21 |
| Oncologist | 16.63 |
| Surgeon | 11.17 |
| Otolaryngologist | 13.40 |
| Neurologist | 16.63 |
| Endocrinologist | 16.63 |
| Geriatrist | 16.63 |
| Urologist | 13.40 |
| Gynecologist | 13.90 |
| Trauma surgeon | 16.63 |
| Dermatologist | 13.40 |
| Nephrologist | 16.63 |
| Ophthalmologist | 16.63 |
| Cardiologist | 16.63 |
| Phono audiologist | 2.96 |
| Nurse | 2.18 |
| Nurse visit | 3.39 |
| Kinesiologist | 2.37 |
| Psychiatrist | 13.57 |
| Psychologist | 5.31 |
| Nutritionist | 2.18 |
| Social worker | 3.39 |
| Therapist | 2.18 |

Source: Author’s calculations based on MLE database[[2]](#footnote-3)

**Table A.3: Other** Prices

|  |  |
| --- | --- |
| **Name/Type** | **Price per hour (US$)** |
| Minimum wage | 2.08 |
| Average wage | 5.59 |
| Median wage | 2.95 |
| Nurse median wage | 3.66 |
| Psychologist median wage | 9.28 |
| Physician median wage | 15.67 |
| Housekeeper median wage | 2.16 |

Source: Author’s calculations based on CASEN Survey[[3]](#footnote-4)

1. SERNAC is the government agency in charge of consumer rights. There are three big pharmacies in Chile, we used two of the biggest ones. [↑](#footnote-ref-2)
2. The MLE database is a database which contains information on the coverage in pesos from the public insurance company, called FONASA. FONASA covers more than 85% of the population. [↑](#footnote-ref-3)
3. CASEN survey is a nationally representative survey. [↑](#footnote-ref-4)
